# Improvement and Neuroplasticity after Combined Rehabilitation to Forced Grasping

**DOI:** 10.1155/2017/1028390

**Published:** 2017-02-06

**Authors:** Michiko Arima, Atsuko Ogata, Kazumi Kawahira, Megumi Shimodozono

**Affiliations:** Department of Rehabilitation and Physical Medicine, Kagoshima University Graduate School of Medical and Dental Sciences, Kagoshima, Japan

## Abstract

The grasp reflex is a distressing symptom but the need to treat or suppress it has rarely been discussed in the literature. We report the case of a 17-year-old man who had suffered cerebral infarction of the right putamen and temporal lobe 10 years previously. Forced grasping of the hemiparetic left upper limb was improved after a unique combined treatment. Botulinum toxin type A (BTX-A) was first injected into the left biceps, wrist flexor muscles, and finger flexor muscles. Forced grasping was reduced along with spasticity of the upper limb. In addition, repetitive facilitative exercise and object-related training were performed under low-amplitude continuous neuromuscular electrical stimulation. Since this 2-week treatment improved upper limb function, we compared brain activities, as measured by near-infrared spectroscopy during finger pinching, before and after the combined treatment. Brain activities in the ipsilesional sensorimotor cortex (SMC) and medial frontal cortex (MFC) during pinching under electrical stimulation after treatment were greater than those before. The results suggest that training under electrical stimulation after BTX-A treatment may modulate the activities of the ipsilesional SMC and MFC and lead to functional improvement of the affected upper limb with forced grasping.

## 1. Introduction

The grasp reflex is a primitive reflex that may reappear in the presence of lesions in the frontal lobe. Involuntary movements and dystonia are well-known features of neurodegenerative diseases in the basal nucleus. They are both distressing symptoms and have specific impacts on the quality of life of patients. There are several effective drugs for involuntary movements, such as L-DOPA, trihexyphenidyl hydrochloride, clonazepam, and botulinum toxin type A (BTX-A) [1, 2]. However, the need to treat or suppress the grasp reflex has rarely been discussed in the literature.

BTX-A is a very effective treatment for spasticity and dystonia in patients with stroke, cerebral palsy (CP), or spinal cord injury (SCI) [3]. To our knowledge, there has been no report on treatment with BTX-A for the grasp reflex.

The objective of this case report is to describe the use of BTX-A treatment accompanied by both repetitive facilitative exercises (RFE) and object-related training under low-amplitude continuous neuromuscular electrical stimulation (NMES) for a patient with the grasp reflex after infarction of the right putamen and temporal lobe. The patient showed functional improvement after BTX-A treatment. The change in brain activity was examined using near-infrared spectroscopy (NIRS).

## 2. Methods/Case Report

### 2.1. Design and Subject

**Figure 1:**
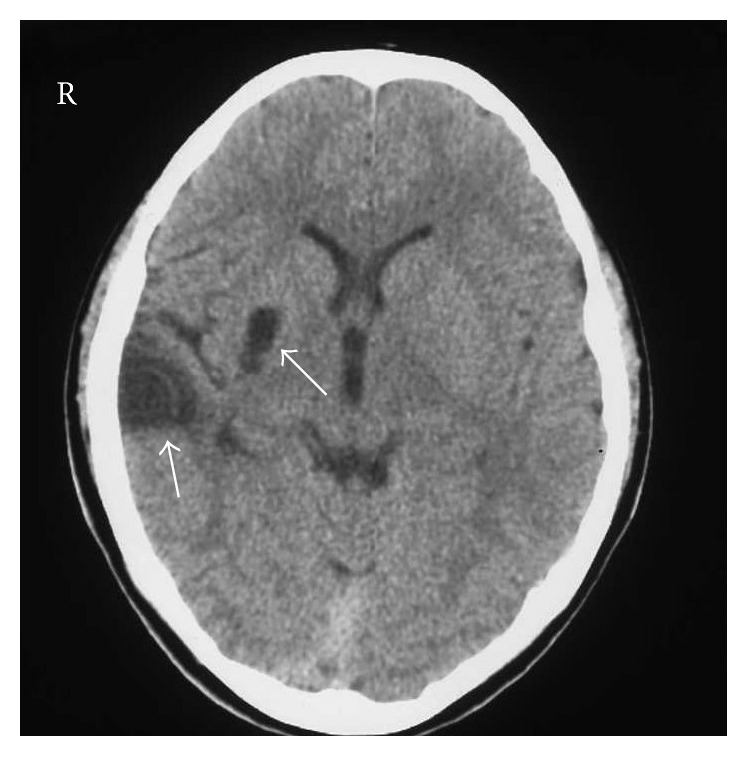
Brain lesion in computed tomography (CT). Low-density areas (arrows) in the right putamen and temporal lobe are observed.

This was a single-patient case study. A 7-year-old boy with right cerebral infarction suffered from left hemiplegia, forced grasping, and unilateral spatial neglect. Brain CT scan revealed cerebral infarction of the right putamen and temporal lobe (Figure 1). Two months later, he was admitted to our hospital for rehabilitation for his hemiplegia. The severity of hemiplegia of the left extremities according to the Brunnstrom stage (BRS) was 2 in the upper limb, 1 in the hand, and 4 in the lower limb. He underwent physical and occupational therapy, including RFE [4, 5] and vibratory stimulation of his palm to reduce forced grasping [6]. After his forced grasping improved, he could grasp and release objects voluntarily. After the three-month admission, he could walk independently and returned to usual school life. The severity of hemiparesis of the left extremities (BRS) was 6 in the upper limb, 4 in the hand, and 6 in the lower limb. One year after discharge, he developed muscular spasticity and rigidity, and these symptoms gradually deteriorated as he grew older. The effect of vibratory stimulation on his forced grasping weakened. About 2 years later he fell and broke his left upper arm, and his left upper limb developed involuntary movement. This involuntary movement consisted of flexing at the elbow, pronate, and he held his left upper limb behind his body because it would rise up suddenly when he walked. Therapeutic trials with amantadine (100 mg/day) and trihexyphenidyl hydrochloride (2 mg/day) showed a slight benefit. He was treated with botulinum toxin for his upper limb when he was 17 years old. With the improvement of spasticity of the upper arm, the grasp reflex and involuntary movement of the upper limb almost completely disappeared. However, the spasticity of the upper limb and improvement of the grasp reflex deteriorated 2 to 3 months later.

He was admitted again for BTX-A treatment at 4 months after his discharge. The severity of left hemiparesis BRS was 5 in the upper limb, 3 in the hand, and 5 in the lower limb. Forced grasping and involuntary movement of the upper limb were so remarkable that he could not use tools with his left hand. The Simple Test for Evaluating Hand Function (STEF) score on admission was only 8 points. The STEF was designed to evaluate the speed of manipulation (catching or pinching objects and carrying them) of objects (10 different shapes and sizes) using an upper limb [7]. The maximum number of points is 100. The Modified Ashworth Scale (MAS) of his hemiparetic upper limb was 2~3 in the upper limb and 3 in the fingers. The MAS is an established and reliable tool which uses a 6-point scale (0, 1, 1+, 2, 3, 4) to score the average resistance to passive movement for each joint [8]. MAS 0 indicates “no increase in muscle tonus.” MAS 4 indicates “affected part(s) rigid in flexion or extension.”

### 2.2. Interventions

**Figure 2:**
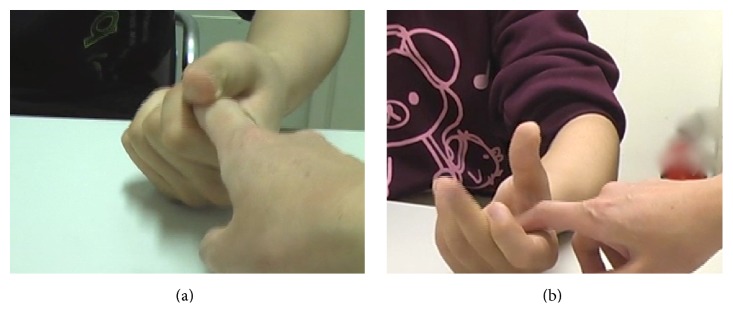
Improvement of the patient's grasp reflex. (a) The patient could not open his hand when the examiner inserted his index finger into the patient's palm before BTX-A treatment. (b) The patient could open his hand when the examiner elicited the grasp reflex after BTX-A treatment.

The patient was treated by the injection of 200 units (U) of BTX-A (Botox; Allergan, Irvine, CA, USA) under electromyography (EMG) guidance soon after admission. BTX-A was injected into the following muscles: left biceps (50 U), flexor carpi radialis (25 U), flexor carpi ulnaris (25 U), flexor digitorum superficialis (35 U), flexor digitorum profundus (25 U), flexor pollicis longus (20 U), flexor pollicis brevis (10 U), and adductor pollicis (10 U), respectively. One week after the BTX-A injections, the spasticity of the left upper limb and the fingers improved and the grasp reflex and involuntary movement nearly disappeared (Figure 2). He could grasp and release objects, but the muscle was still weak. The MAS of the upper limb was 1~2, and those of the fingers were 2.

**Figure 3:**
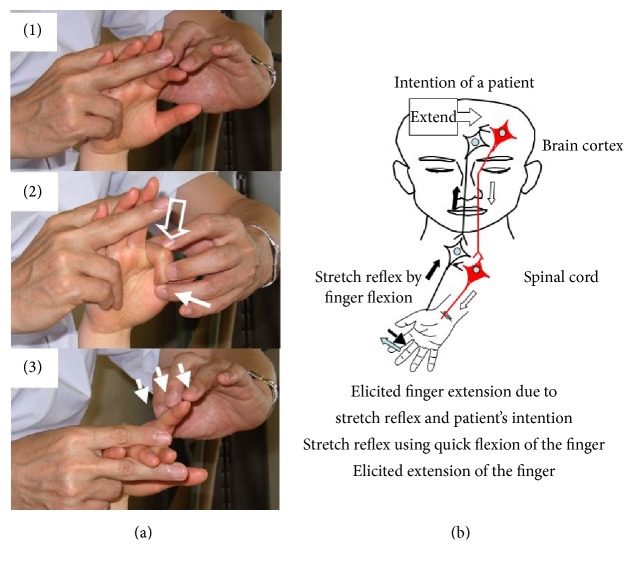
Hypothesized mechanism of a newly designed facilitation exercise for the hemiplegic upper limb and fingers. (a) Facilitation for extension of the isolated finger is performed as follows: (1) the finger is quickly flexed by the therapist; this elicits the stretch reflex; (2) the therapist instructs, “Extend” and pushes the proximal phalanx to flex the metacarpophalangeal (MP); and (3) the therapist applies slight resistance against finger extension to maintain extension of the finger. The thick arrow and thin arrow indicate manipulation to induce the stretch reflex and light touch (resistance) to maintain the *α*-*γ* linkage, respectively. (b) Descending motor tracts related to the patient's intention to move will respond to excitation of the patient's intention when these neural tracts are excited to a sufficient excitation level by facilitation techniques and result in the realization of movements by the patient's intention. Modified from Figure 1 in Kawahira et al. (2010).

RFE were applied after the BTX-A injections. RFE were designed to elicit and maintain movements isolated from synergy, including the movement of each isolated finger using a stretch reflex, skin-muscle reflex, and alpha-gamma linkage. The hypothesized mechanism of the newly designed facilitation exercises for the fingers is shown in Figure 3 [9]. In patients with hemiparesis, descending motor tracts involved in movements intended by the patient do not discharge because of a low excitation level. If the excitation level in these neural circuits is adjusted and excitation is timed to discharge by the facilitation techniques, upon neuronal excitation of the patient's intention which originates in the prefrontal/premotor cortex, these neural circuits would discharge and realize movement intended by the patient.

**Figure 4:**
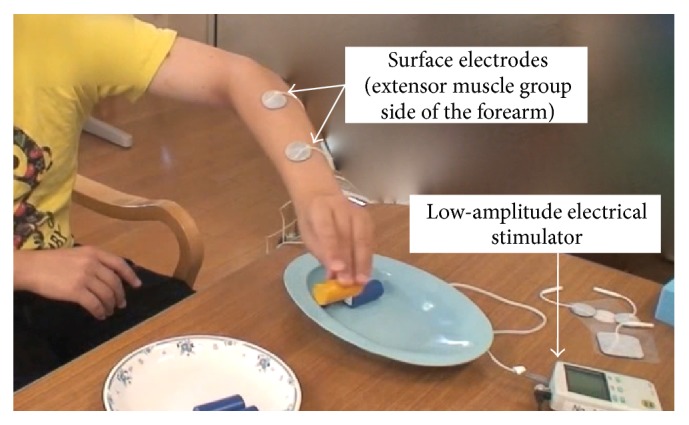
Effect of continuous electrical stimulation on the patient's ability to carry 5 pegs. The electrodes were attached to the extensor side of the forearm. The patient was instructed to carry each peg from one dish to another dish. The length of time required to carry the 5 pegs improved from 19.4 s without continuous electrical stimulation to 14.5 s with continuous electrical stimulation.

When low-amplitude continuous NMES was applied to the left wrist and finger extensor muscles with surface electrodes, he could grasp and release objects easily (Figure 4). The stimulation pulse was a symmetrical biphasic waveform, with a pulse width of 250 *μ*s and frequency of 20 Hz. The intensity of the electrical current was adjusted to produce slight contraction of the target muscle without inducing obvious limb/joint movement while the patient remained at rest and was subjectively comfortable. He repeated the training to grasp and release the object under NMES. RFE were applied under NMES [10] for about two weeks.

The function of the hand and upper limb improved over two weeks from a STEF score of 8 to 31 points. The severity of hemiparesis of the hand improved from BRS 3 to 5 when he was discharged.

### 2.3. Functional NIRS (fNIRS)

The effect of BTX-A treatment combined with both RFE and object-related training under low-amplitude continuous NMES (BTX-A treatment combined with NMES) was examined by using NIRS. NIRS was performed before and after BTX-A treatment combined with NMES.

**Figure 5:**
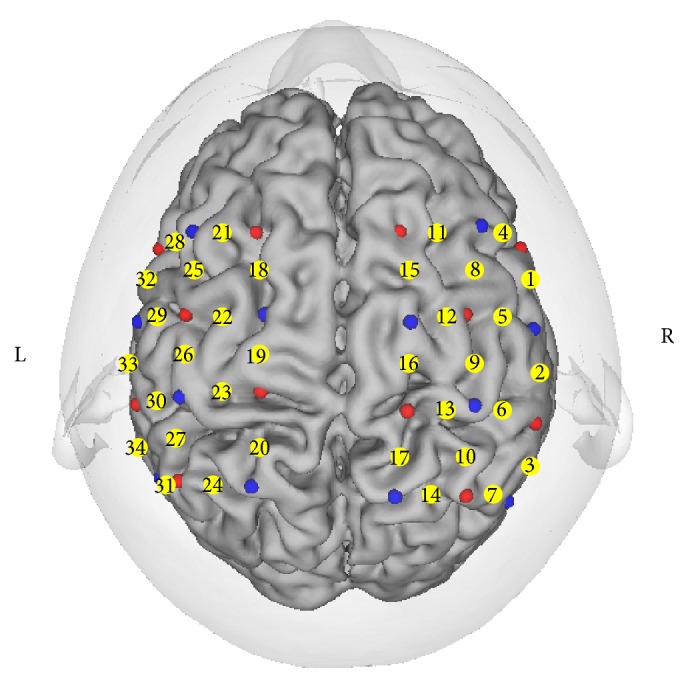
Projection of the probes and the channels onto the brain surface, using MRI data and a 3D position detector. Red: source, blue: detector, and yellow: channel.

Thirty-four channels of a 52-multi-channel NIRS device (OMM-3000/16, Shimadzu Co., Kyoto, Japan) were placed in 2 reticular patterns on both sides around the sensorimotor cortex (SMC) of the patient. The bottom row of channels was set parallel to the T3-F7 (left) and T4-F8 (right) line, since C3 and C4 should be covered by a total of 3 or 4 channels. T3, T4, F7, F8, C3, and C4 are defined in the International 10–20 system of electroencephalography. Each channel measures the fluctuation of the concentration of oxygenated hemoglobin ([oxy-Hb]) and the concentration of deoxygenated hemoglobin ([deoxy-Hb]) using 3 wavelengths (780 nm, 805 nm, and 830 nm) according to the Beer-Lambert law. These [oxy-Hb] values reflect not only the hemoglobin density but also thepath length of light. Each channel is configured by a pair of source/detector probes, which are separated by a distance of 30 mm. The channel is supposed to be set at the midpoint between the two probes 20~30 mm under the scalp (see Figure 5).

The source probe emitted light sequentially to avoid cross-talk noise, and the sampling time was adjusted to 0.1 s which was sufficiently fast to measure the fluctuation.

**Figure 6:**
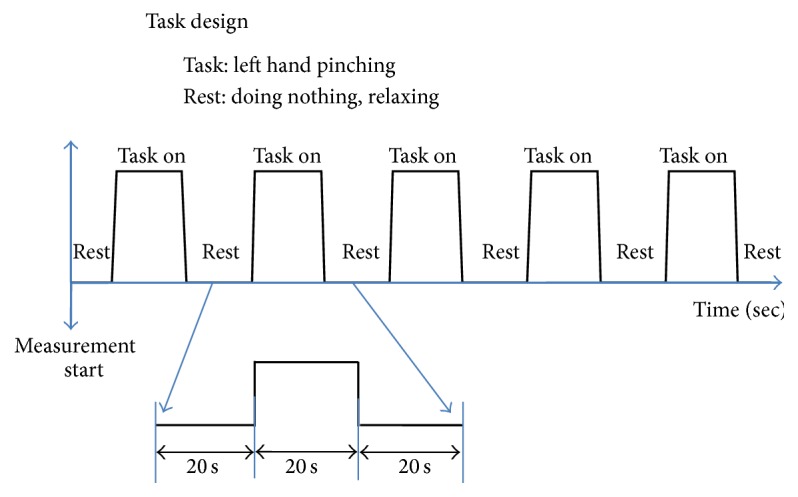
The procedure of the pinching task. The patient was instructed to relax for 10 s, and then the operator ordered the patient to start pinching with the left thumb and index finger every 2 s at the operator's command. After 20 s of pinching, the patient was instructed to relax for another 10 s: 5 cycles of 10 s rest, 20 s pinching task, and 10 s rest were performed for averaging. Therefore, there is an interval of 20 s between 2 pinching tasks.

The time course data were acquired at each channel during the pinching test. The patient sat on a chair during the experiment. The patient was instructed to relax for 10 s, and then the operator ordered the patient to start pinching with the left thumb and index finger every 2 s at the operator's command. After 20 s of pinching, the patient was instructed to relax for 10 s; 5 cycles of this 10 s rest, 20 s pinching task, and 10 s rest were performed for averaging (Figure 6). Two conditions were investigated: (1) voluntary finger pinching and (2) voluntary finger pinching with continuous electrical stimulation of the wrist extensor muscles.

The statistical significance of differences in hemodynamic responses was assessed by a general linear model (GLM); the time course of Δ oxy-Hb was correlated with the design matrix using a boxcar function. The statistical significance of differences in all GLM analyses was based on an adjusted alpha level of less than 0.05, which corresponded to *T* values greater than 2.3 [11].

## 3. Results

The effect of BTX-A treatment combined with NMES on brain activity was examined by NIRS.

**Figure 7:**
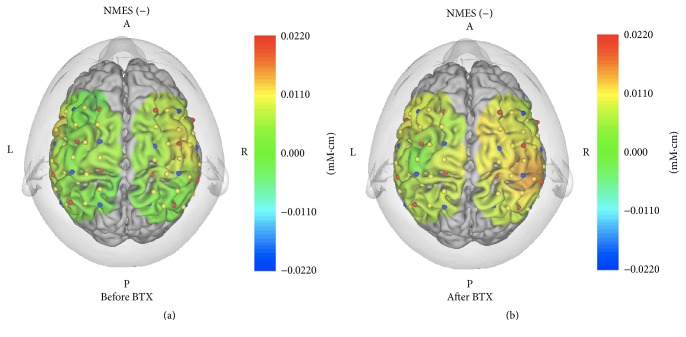
Three-dimensional maps of hemodynamic responses (changes in the oxy-Hb concentration) in the brain while the patient performed finger pinching with the left hand before BTX-A treatment (a) and after BTX-A treatment (b). There was an increase in activation in the ipsilesional SMC after BTX-A treatment compared with that before BTX-A treatment.

There was an increase in activation (increase in [oxy-Hb]) in the ipsilesional SMC after BTX-A treatment combined with NMES compared with that before BTX-A treatment (Figures 7(a) and 7(b)).

**Figure 8:**
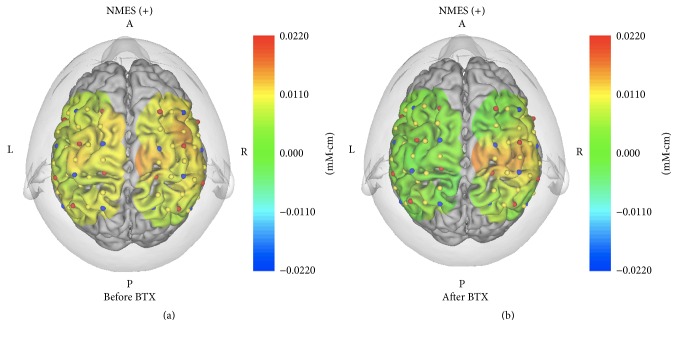
Three-dimensional maps of hemodynamic responses (changes in the oxy-Hb concentration) in the brain while the patient performed pinching with the left hand under continuous electrical stimulation of the extensor side of the forearm before BTX-A treatment (a) and after BTX-A treatment (b). There was activation in both SMC and ipsilesional PFC before BTX-A treatment, and the activation area was more localized at the ipsilesional SMC after BTX-A treatment.

When continuous NMES was applied to the extensor side of the forearm during pinching, there was activation in both SMC and ipsilesional prefrontal cortex (PFC) before BTX-A treatment combined with NMES (Figure 8(a)). The activation area was more localized at the ipsilesional SMC after BTX-A treatment (Figure 8(b)).

In a comparison of the activation area after BTX-A treatment combined with NMES, the activation area with continuous electrical stimulation of the extensor side of the forearm (Figure 8(b)) was smaller than that without continuous electrical stimulation (Figure 7(b)).

## 4. Discussion

The grasp reflex and involuntary movement after cerebral infarction improved with a decrease in spasticity by BTX-A treatment combined with both RFE and object-related training under continuous NMES. NIRS detected an increase in blood flow in the right cerebral motor cortex during left hand pinching after BTX-A treatment combined with NMES. More regional activity of the right motor cortex was detected during continuous electric muscle stimulation of the extensor side of the forearm.

The grasp reflex can be elicited in neonates and early infants as a result of insufficient control of the spinal mechanism by the immature brain, but the reflex gradually disappears as the infant grows, due to increased inhibition accompanying brain maturation [12]. Adult patients with lesions in the frontal lobes sometimes exhibit a grasp reflex of the hands and feet [12]. The reappearance of each of these reflexes in adults is attributed to the release of the spinal reflex center from the disturbed higher brain mechanism, suggesting that these reflexes are only inhibited and not lost after infancy [13].

In a study by De Renzi and Barbieri [14], the palmar grasp reflex was elicited in 21 (66%) of 32 patients with a medial frontal lesion and in 8 (26%) of 30 patients with a lateral frontal lesion. On the other hand, a small percentage of patients with a deep lesion including the basal ganglia without frontal cortical damage were reported to exhibit a positive palmar grasp reflex [14], and the extension of a supplementary motor area (SMA) lesion into more lateral regions of area 6 may increase the strength of the grasp reflex [15].

In our patient, it seems that inhibitory stimuli from upper brain structures were obstructed by lesion of the right putamen to release the spinal grasp reflex center.

A major role of the basal ganglia could be to achieve a balance between excitatory and inhibitory thalamocortical influences. The basal ganglia appear to “gate” sensory input at various levels [16]. There is some indirect evidence that this sensory gate for motor control is lost in dystonia [17]. In our patient, the putaminal lesion could have altered this delicate balance, leading to distorted afferent cortical information. Before BTX-A treatment, electrical stimulation of the left forearm activated a wide area in both cerebral cortices on NIRS. It is possible that this was due to abnormal sensorimotor integration [18], and the patient performed pinching with great effort.

Intramuscular injection of BTX-A was performed to treat muscle spasticity and dystonia. The therapeutic effects of BTX-A seemed to be due not only to partial denervation of extrafusal muscles but also to fusimotor denervation of intrafusal fibers that tonically control the sensitivity of spindle sensory afferents [19–21]. Consequently, BTX-A may induce changes in the alpha-gamma linkage of volitional control [22]. In addition, BTX-A applied periphery may directly induce central plasticity in spinal cord via retrograde axonal transport, especially at high doses [21]. It is not yet clear that this direct central effect is induced at therapeutic dose such as in our case report.

Continuous electrical stimulation of the wrist and finger extensor muscles after BTX-A treatment could also inhibit antagonistic muscles such as the flexor muscles of the wrist and finger. Brain activity in the ipsilesional SMC and MFC areas was more improved with continuous electrical stimulation while pinching, as examined by NIRS. Furthermore, activities in the contralesional hemisphere during continuous electrical stimulation appeared to be suppressed after BTX-A treatment (Figure 8(b)). This suppression might also contribute to the present recovery because activities in contralesional M1 sometimes disturb motor recovery by abnormal interhemispheric interactions during voluntary movement of the paretic hand [23–25].

In our patient, we considered that the frontal lobe did not suppress the grasp reflex or involuntary movement induced by sensory input to the left hand. However, BTX-A treatment combined with NMES improved voluntary movements. This improvement may have been due to not only a decrease in peripheral spasticity, but also a decrease in the excitement from the muscle spindle to the afferent nerve fibers to the spinal cord, and this indirectly influenced the cerebral cortex and improved the cerebral balance to make it easy to perform voluntary movement. Electrically mediated repetitive movement may facilitate the neuroplasticity of motor learning.

## 5. Conclusions

The grasp reflex of the hemiplegic left upper limb of a patient who suffered infarction of the right putamen and temporal lobe was significantly improved by BTX-A treatment combined with both RFE and object-related training under NMES. This improvement of the grasp reflex by BTX-A consisted of not only the improvement of spasticity but also the improvement of motor control of finger movement. Further research on BTX-A treatment combined with NMES is needed in patients with the grasp reflex that is not improved with ordinary treatment.
